# Methods of Selenium Application Differentially Modulate Plant Growth, Selenium Accumulation and Speciation, Protein, Anthocyanins and Concentrations of Mineral Elements in Purple-Grained Wheat

**DOI:** 10.3389/fpls.2020.01114

**Published:** 2020-07-21

**Authors:** Qing Xia, Zhenping Yang, Yang Shui, Xiaoli Liu, Jie Chen, Shahbaz Khan, Jianming Wang, Zhiqiang Gao

**Affiliations:** College of Agriculture, Shanxi Agricultural University, Taigu, China

**Keywords:** selenium biofortification, purple-grained wheat, anthocyanins, organic selenium, elements

## Abstract

Selenium (Se) is an essential micronutrient for human health. Deficiency and suboptimality of Se in human populations are a potential health risk. The reduction of such health risk by biofortification of crops, particularly in wheat has drawn much attention, especially for color-grained wheat as it is rich in anthocyanins and can be used as a major source of antioxidants in diet. Herein, a two-year field study on the purple-grained wheat cultivar (202w17) and common wheat cultivar (Shannong 129) was conducted with soil application (SeS) and foliar spray (SeF) of selenium. Results showed that the SeS increased shoot dry weight and grain yield. Both SeS and SeF enhanced the concentration of organic Se, but the higher concentration of organic Se in the grain of two cultivars was observed in SeF in comparison with SeS. The concentration of organic Se in the grain of 202w17 treated with SeF was approximately 1.5-fold of that in Shannong 129 with SeF. The analysis of Se accumulation in different parts of the plant revealed that 202w17 accumulated more Se in shoots and grain than Shannong 129, and 202w17 had also higher levels of total protein, total free amino acids and anthocyanin in grain than Shannong 129. In addition, SeF significantly increased the concentrations of zinc (Zn), calcium (Ca), magnesium (Mg) in both cultivars, but decreased the concentration of chromium (Cr), cadmium (Cd) and lead (Pd), which phenomenon was more significant in 202w17. Our results indicate that SeS increases plant growth, leading to higher grain yield in two cultivars tested. The purple-grained wheat (202w17) could accumulate more Se in grain and have a higher concentration of orgainic Se in grain than the common wheat (Shannong 129).

## Introduction

Selenium (Se) is an essential micronutrient for human health, and it plays a critical role in antioxidant, anti-cancer, anti-bacterial, and antiviral activities (Lyons, 2018). For higher plants, Se has been shown to be beneficial under stress conditions (Jóźwiak and Politycka, 2019), but not essential for normal functions. Soils low in Se are found for 72% of the total area in China (Gao et al., 2011). Se concentrations in most food crops produced in China are usually less than 60 μg kg^−1^ (Williams et al., 2009). It has been estimated that more than 70 million people in China have potential risk of Se deficiency (Gao et al., 2011). Recently, dietary Se has been recognized to be safer than the supplementation of inorganic Se (Liu et al., 2012). Therefore, cereal crops widely consumed become a target of Se biofortification to increase Se status of population (Fairweather-Tait et al., 2011). Biofortification with agronomic approaches shows advantage in effectiveness and sustainability over direct supplementation of inorganic Se.

Wheat is a major staple crop worldwide. Although wheat plants have a low capacity to assimilate Se, they have a relatively high ability to transport Se from straw to seed (Wang et al., 2017). Therefore, Se biofortification in wheat with agronomic approaches has been shown to be practical. Foliar and soil application of Se are two main practices for agronomic biofortification of Se (Shalaby et al., 2017). Generally, foliar application is more effective in increasing Se concentrations in wheat grains than soil application (Nawaz et al., 2015). Se-enriched wheat grains can be used as a dietary source of Se to reduce human health risk of Se deficiency. Recently attention has been paid to color-grained wheat in China and other Asian countries as it is rich in anthocyanin (Guo et al., 2013).

Color-grained wheat genotypes (blue, purple, and black), which differ from the common wheat ones (red or white), contain different types of anthocyanin (antioxidant pigments) in the aleurone and pericarp (Tian et al., 2018). Anthocyanins display various biological activities including antioxidant, anti-inammatory, antimicrobial and anti-carcinogenic activities, and are non-toxic for human consumption (Varga et al., 2013). The color-grained wheat cultivars contain other natural compounds which are beneficial to human health. The color-grained wheat with high nutritional value and antioxidant pigments has the potential to make value-added, functional flour products (Pasqualone et al., 2015). Previous studies have also found that protein and amino acid content in some of color-grained wheat cultivars are at least 7% higher than those in common wheat (Tian et al., 2018). In addition, color-grained wheat cultivars contain other natural compounds which are beneficial to human health. The color-grained wheat with high nutritional value and antioxidant pigments has the potential to make value-added, functional flour products (Pasqualone et al., 2015). However, it is not clear how different methods of Se application affect the concentrations of Se, other mineral elements and health-promoting phytochemicals in color-grained wheat.

The objectives of this study were i) to examine the effect of soil and foliar application of Se on dry weight, grain yield, Se accumulation in different parts of the plant, and Se species, anthocyanin, protein and amino acids, and mineral elements of grain in the purple-grained wheat cultivar (202w17), and ii) to make comparison of such an effect with the common wheat cultivar (Shannong 129).

## Materials and Methods

### Experimental Site

**Figure 1:**
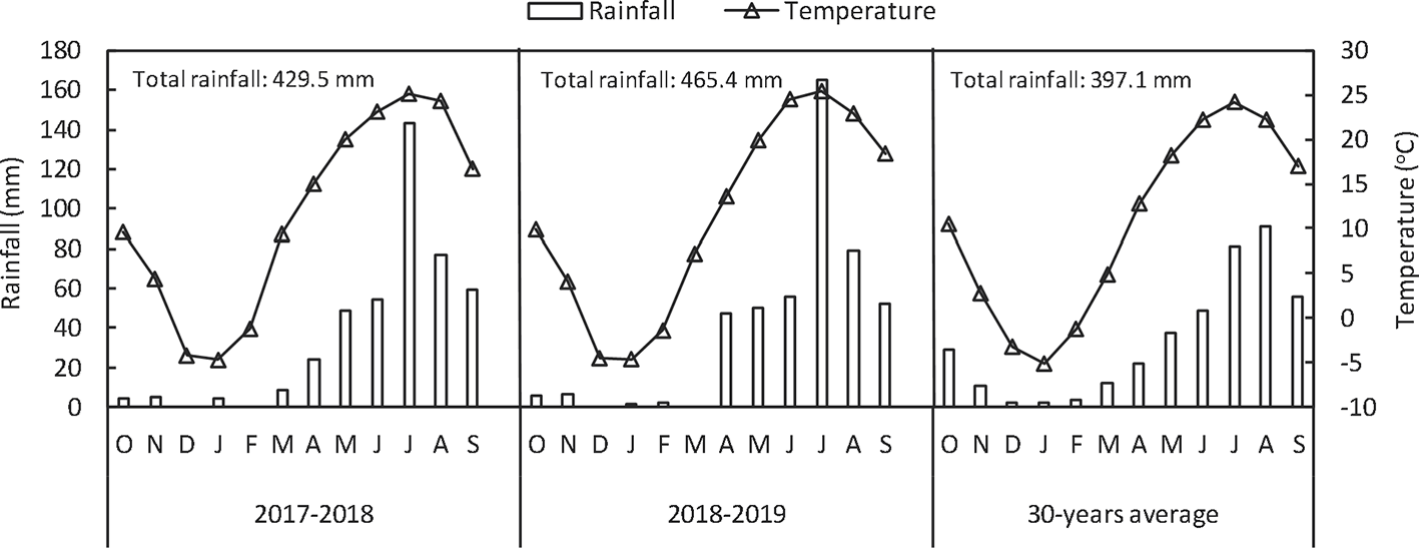
Monthly and annual rainfall and mean temperatures in 2017–2018, 2018–2019 and in an average year from a 30-year period at Taigu (China).

Field experiments were conducted for two seasons (2017–2018 and 2018–2019) in Wujiazhuang village, Taigu, Shanxi Province, China (37°26’ N, 112°31’ E, 802 m above the sea level). The experimental field soil was classified as silty clay loam (Chinese soil taxonomy). The precipitation and temperature at the experimental site from October, 2017 to June, 2019 are shown in **Figure 1**. The chemical properties of soil (0–20 cm) were: organic matter, 19.96 g kg^−1^; available nitrogen, 59.51 mg kg^−1^; available phosphorous, 4.51 mg kg^−1^; available potasssium, 195.86 mg kg^−1^ and total Se, 0.28 mg kg^−1^. Soil organic matter was determined with the modiﬁed Walkley–Black method (Wang et al., 2016); available nitrogen was measured by the alkali-hydrolyzable proliferation method; available P (Olsen-P) was measured by extraction with 0.5 mol L^−1^ NaHCO_3_ followed by colorimetric measurement of P using the molybdate-ascorbic acid method; available K by extraction with 1 mol L^−1^ ammonium acetate was analyzed by a ﬂame photometer; and Se concentrations were measured by inductively coupled plasma mass spectrometer (ICP-MS, Agilent 7700x; Agilent Technologies, USA).

### Experimental Design

**Figure 2:**
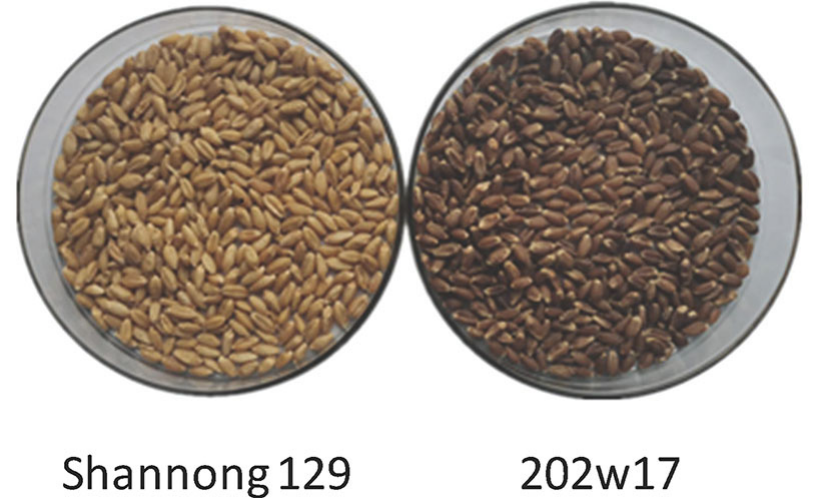
The phenotype of two wheat cultivars.

The experiment was set up as a split-plot design with three replicates. Main plot treatments were two wheat varieties, 202w17 (purple-grain) and Shannong 129 (normal red-grain) (**Figure 2**). Subplot treatments were the control with no Se application (Se0), soil application of Se (SeS) and foliar spray of Se (SeF). Our previous work with a range of Se rates from 0 to 115 g Se ha^−1^ showed that foliar spray of 37.5 g Se ha^−1^ could increase Se concentrations up to 290 μg kg^−1^ in the grains of wheat cultivars including purple-grained wheat (Xia et al., 2019). The Se concentration of 300 μg kg^−1^ in wheat grain is considered to be safe for humuan consumption (Lyons, 2010). Therefore, Se of 37.5 g ha^−1^ was applied as the form of Se^4+^ (Se-enriched solid fertilizer and Se-enriched nutrient solution) for both soil and foliar applications. The total Se concentration of Se-rich solid fertilizer used in the experiment is 50 mg kg^−1^, and the Se-enriched liquid fertilizer used is 10 mg ml^−1^. Soil application of Se-enriched solid fertilizer was conducted before sowing. For foliar spray of Se-rich nutrient solution, a hand compression sprayer was used to spray 750 L ha^−1^ solution (50 mg Se L^−1^) to wheat leaves in the SeF treatment at flowering, and the same amount of distilled water in the control (Se0). No precipitation occurred at least seven days after foliar spray. Each plot (3 m × 8 m) was applied with 750 kg ha^−1^ of the compound fertilizer containing N 18%, P_2_O_5_ 22% and K_2_O 5% prior to planting. Before sowing, the compound fertilizer and remaining wheat stubble (20–30 cm) were ploughed to 25–30 cm depth using a rotavator in the early to middle of July as deep ploughing. Wheat seeds were sown at a density of 225 kg ha^−1^ and drilling sowing was applied by using the planting machine (2BMF-12/6) with row spacing of 20 cm. Weed control was performed by hand pulling and the application of 2,4-dichlorophenoxyacetic acid at bolting. No additional water or fertilizer was applied during plant growth. Sowing dates were October 15, 2017 and October 6, 2018, and plants were harvested on June 19, 2018 and June 17, 2019.

### Sample Preparation and Measurments of Se and Se Forms and Elements

FAt harvest, ten plants per plot were randomly sampled for Se analysis. For each plant, the soil was dug out at 15 cm radius around the plant, and the soil loosely bound to roots was shaken off. Then the plant was separated into roots, stem + leaves, glume + spike axis and grain. After that, roots, stem + leaves and glume + spike axis were carefully washed with tap water to remove soil and other contaminants, followed by quickly rinsing three times with deionized water. The plant parts were oven-dried at 65°C until weight was constant, and ground to powder with motorized pulverizer (FZ102, Shanghai, China). Then plant powder was passed through a 0.15 mm sieve and stored in ziplock bags for the analysis of Se. Grains were ground to flour using a motorized pulverizer (HCP 100, Zhejiang, China), and the flour was sieved through a 0.15 mm sieve and sealed in ziplock bags for the analysis of total Se and Se forms, total protein, free amino acids and anthocyanins. In addition, representative plants of three rows (1.1 m in length) were hand-harvested at maturity from each plot, placed in a mesh bag, and air-dried for total grain yield.

For the measurement of Se and other mineral elements, plant and flour samples (100 mg) were digested by using a HNO_3_–H_2_O_2_ mixture (4:1 ratio v/v) in an alimentary furnace reaction system (LWY84B, China), and the digestion solution was used for the measurement with ICP-MS as described above. For quality assurance, blank and standards (GBW(E) 080215 for Se, and GNM-M22809-2013 for the other mineral elements) were included in the measurements. To measure different Se forms, flour samples (200 mg) were extracted in 5 ml Tris–HCl with sonication for 30 min. Then, 50 mg cellulose enzyme, 0.4 ml proteinase K and 0.4 ml proteinase XIV were added to the solution, and the solution was incubated in an air bath thermostat (50 °C, 250 r/min) for 48 h. After that, the solution was centrifuged at 1,000 r/min, 4 °C for 30 min, and the supernatant was collected and filtered through 0.22 μm membrane. The filtrate was used to determine different Se forms in grain by high performance liquid chromatography-atomic fluorescence analyzer (AFS-9230-SPA-10-LC-20AB, Beijing Jitian, China).

### Measurements of Protein, Total Free Amino Acids, and Anthocyanins

Total grain protein was measured with the Kjeldahl nitrogen method (Malavolta et al., 1997). The concentrations of anthocyanins were determined with the pH differential method (Giusti and Wrolstad, 2001). For the measurements of total free amino acids, flour (5 g) was extracted with 10 ml HCl (6 mol L^−1^) and analyzed as described by Cocking and Yemm (1954).

### Statistical Analysis

SAS software (SAS 8.0, USA) was used for analysis variance by using the split-plot design model. Least Signiﬁcance Diﬀerence (LSD) tests at P = 0.05 was used for comparison of means.

## Results

### Effects of Se Application on Shoot Dry Weight and Grain Yield in Wheat During Two Seasons

**Figure 3:**
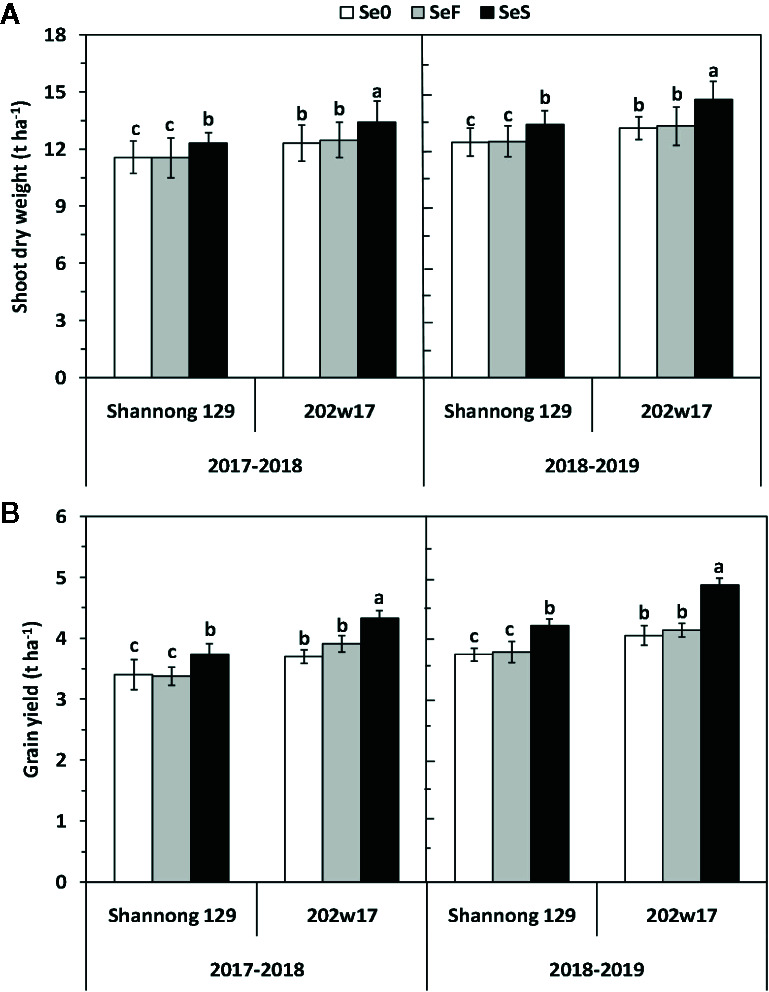
Effects of Se application on shoot dry weight and grain yield in wheat plant during two seasons. Shoot dry weight **(A)** and grain yield **(B)**. Se0, no Se control; SeF, foliar spray of Se; SeS, soil application of Se. Means and standard errors of three replicates are presented. The means are not significantly different within a given season when followed by the same lowercase letter using LSD at P < 0.05.

Soil application of Se (SeS) significantly increased shoot dry weight and yield in both purple-grained wheat (202w17) and common wheat (Shannong 129) relative to the control at Se0 in two seasons, but foliar spray of Se (SeF) had no significant effect on shoot dry weight and grain yield (**Figures 3A, B**). The shoot dry weight of 202w17 treated with SeS was 10.1 and 9.0% higher on average in two seasons than that of 202w17 with Se0 or SeF, respectively (**Figure 3A**), and the yield of 202w17 treated with SeS was 19.0 and 14.6% higher on average in two seasons than that of 202w17 with Se0 or SeF, respectively (**Figure 3B**). Notably, 202w17 had 7.7% higher shoot dry weight on average of three treatments in two seasons than Shannong 129 (**Figure 3A**), and 12.4% higher yield than Shannong 129 (**Figure 3B**). These results indicate that the soil application of Se could increase plant growth and grain yield. The multiple variance analysis showed that cultivar and Se application methods had the largest effect on shoot dry weight and grain yield during two seasons (**Table 1**).

**Table 1:** F value and significance for shoot dry weight, yield, Se concentrations, SeMet, and SeCys2.

Year	Source of variance	Shoot dry weight	Yield	Se concentrations				SeMet	SeCys2
				Roots	Stalk+leaves	Glume+spike axis	Grain		
2017-2018	Cultivar	84.82*	27.60*	0.01	2.68	11.57	14.84	23.95*	1.03
	Fertilization method	33.74*	10.49*	21.93*	10.38	36.11*	37.81*	55.38*	3.00
	Cultivar×Fertilization method	14.53**	9.33**	45.45***	125.65***	74.12***	31.29***	4352.48***	107.77***
2018-2019	Cultivar	28.07*	14.70*	0.01	2.68	9.67	17.71	23.56*	0.55
	Fertilization method	19.30*	12.59*	21.98*	10.39	31.80*	44.74*	55.95*	2.07
	Cultivar×Fertilization method	83.19***	16.15*	49.10***	125.97***	120.00***	31.00***	9297.54***	137.94***

*indicates a highly significant difference at P < 0.05; **indicates a highly significant difference at P < 0.01; ***indicates a highly significant difference at P < 0.001.

### Effects of Se Application on Dry Matter and Se Concentrations of Different Parts in Wheat Plant During Two Seasons

The effects of Se application on dry matter of different parts in wheat plant (**Figure S1**) were similar to those of shoot dry weight (**Figure 3**). SeS significantly increased dry matter of all parts of the plant relative to either SeF or Se0 in both wheat cultivars and in two seasons except for the glume + spike axis (**Figures S1A, B**). In contrast, SeF had no significant effect on dry matter of any part of the plant relative to Se0 in either wheat cultivar or season (**Figures S1A, B**).

**Figure 4:**
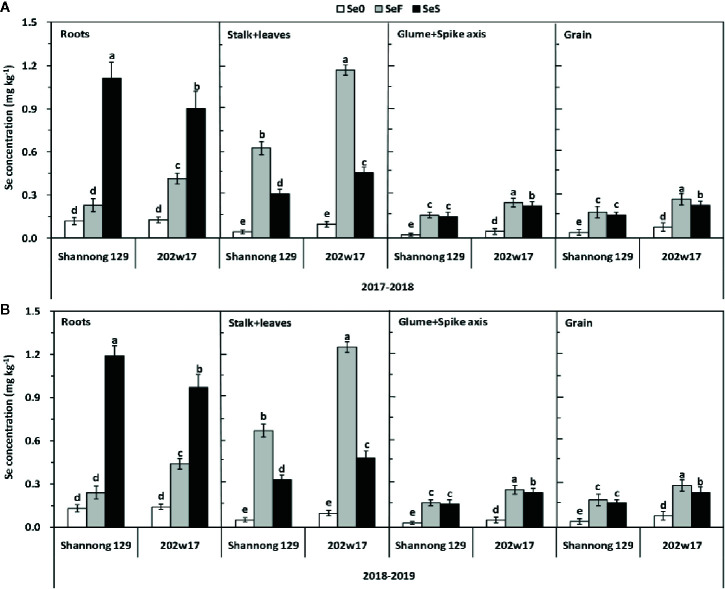
Effects of Se application on Se concentrations of different parts of wheat plants during two seasons. 2017–2018 season **(A)** and 2018–2019 season **(B)**. Se0, no Se control; SeF, foliar spray of Se; SeS, soil application of Se. Means and standard errors of three replicates are presented. The means are not significantly different within a given season when followed by the same lowercase letter using LSD at P < 0.05.

The effects of Se application on the concentration of Se in different parts of the plant differed significantly between two methods and between two wheat cultivars except for the glume + spike axis and grain in Shannong 129 (**Figure 4**). The concentration of Se in the roots of Shannong 129 with SeS was 23.6% higher on average in two seasons than that in 202w17 with SeS, whereas the concentration of Se in the roots of 202w17 with SeF was 81.1% higher on average in two seasons than that in Shannong 129 with SeF (**Figures 4A, B**). The concentration of Se in the stalk + leaves of 202w17 with either SeF or SeS was also higher than that in Shannong 129 (**Figure 4**). 202w17 with SeF or SeS had 86.8 and 46.8% higher Se concentration of stalk + leaves than that in Shannong 129 on average during two seasons, respectively (**Figures 4A, B**). In addition, the concentrations of Se in the glume + spike axis and grain of 202w17 with SeS or SeF were higher than those in Shannong 129 (**Figure 3**). The concentration of Se in the grain of 202w17 with SeF was 49.1% higher on average in two seasons than that in Shannong 129 with SeF, and the concentration of Se in the grain of 202w17 with SeS was 41.1% higher on average in two seasons than that in Shannong 129 with SeS (**Figures 4A, B**). The higher accumulation of Se in the roots of 202w17 than that in Shannong 129 when Se was applied as foliar spray indicates that 202w17 can translocate more assimilated Se from shoots to roots *via* phloem. The higher accumulation of Se in the shoots and grain of 202w17 than that in Shannong 129 when Se was applied either to leaves or to soil indicates that 202w17 can translocate more Se from roots to shoots and from shoots to grain than Shannong 129.

### Effects of Se Application on the Concentrations of SeMet and SeCys2 in the Grain of Wheat During Two Seasons

**Figure 5:**
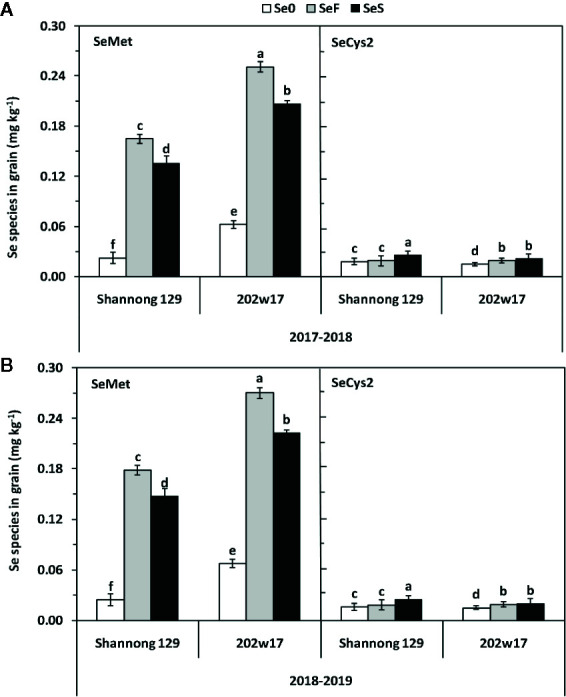
Effects of Se application on the concentrations of SeMet and SECys2 in the grain of wheat during two seasons. 2017–2018 season **(A)** and 2018–2019 season **(B)**. SeMet, selenomethionine; SeCys2, selenocystine; Se0, no Se control; SeF, foliar spray of Se; SeS, soil application of Se. Means and standard errors of three replicates are presented. The means are not significantly different within a given season when followed by the same lowercase letter using LSD at P < 0.05.

By comparing with the spectrumgram of six Se standards, which are selenocystine (SeCys2), Se-methylselenocysteine (MeSeCys), selenite, selenomethionine (SeMet), selenate and seleno-D,L-ethionine, only SeMet and SeCys2 were found in the extracts from grain. SeMet was the major Se species in all samples, which accounts for at least 84.3% of the total grain Se on average in two seasons for either cultivar with SeS or SeF, whereas SeCys2 consisted of 15.7% or less of the total grain Se (**Figures S2A, B**). Se application methods and cultivars showed significant differences in the concentrations of SeMet and SeCys2 (**Figure 5**, **Table 1**). The SeMet concentrationin the grain treated with SeF were 21.3 and 389.9% higher on average in two cultivars and in two seasons than that with SeS or Se0, respectively. The SeMet concentration in the grain of 202w17 was 52.1% higher on the average of two methods of Se application in two seasons than that in Shannong 129 (**Figures 5A, B**). In contrast, the SeCys2 concentration in Shannong 129 with SeS was significant higher on average in two seasons than that in Shannong 129 with SeF, as well as that in 202w17 with SeS or SeF (**Figures 5A, B**). No difference in the SeCys2 concentration was observed in 202w17 between SeS and SeF (**Figures 5A, B**).

### Effects of Se Application on Total Protein Content and Total Free Amino Acids Concentration in the Grain of Wheat During Two Seasons

**Figure 6:**
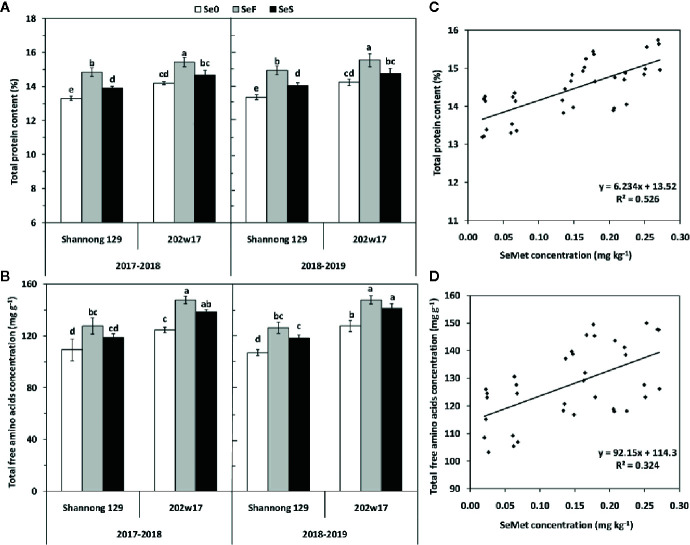
Effects of Se application on total protein content and total free amino acids concentration in the grain of wheat during two seasons. Total protein **(A)** and total amino acids **(B)**. The relationship between the SeMet concentration and total grain protein content **(C)** or the total concentration of free amino acids **(D)**. Se0, no Se control; SeF, foliar spray of Se; SeS, soil application of Se. Means and standard errors of three replicates are presented. The means are not significantly different within a given season when followed by the same lowercase letter using LSD at P < 0.05.

The higher concentrations of SeMet in the grain of 202w17 than that in Shannong 129 prompt us to examine levels of total protein and total free amino acids in grain. Both soil and foliar application of Se significantly increased total protein content of grain in two cultivars and in two seasons relative to Se0 except for 202w17 with SeS (**Figure 6A**). The total protein content in the grain of 202w17 with SeF and SeS was 4.0 and 5.3% higher on average in two seasons than that in Shannong 129, respectively. SeS increased total protein content in grain relative to Se0 only observed in Shannong 129, and it increased by 4.8% on average in two seasons (**Figure 6A**). For total free amino acids in grain, 202w17 with SeS and SeF had higher concentrations than Shannong 129 in two seasons, but no significant difference was observed between SeF and SeS in either cultivar (**Figure 6B**). The regression analysis of all samples in two seasons showed that the SeMet concentration in grain is positively correlated with the total protein content (P < 0.01; **Figure 6C**) and the concentration of total free amino acids in grain (P < 0.01, **Figure 6D**), but not with grain weight (r = 0.2145, P > 0.05).

### Effects of Se Application on Anthocyanin and Elements Concentrations in the Grain of Wheat During Two Seasons

**Figure 7:**
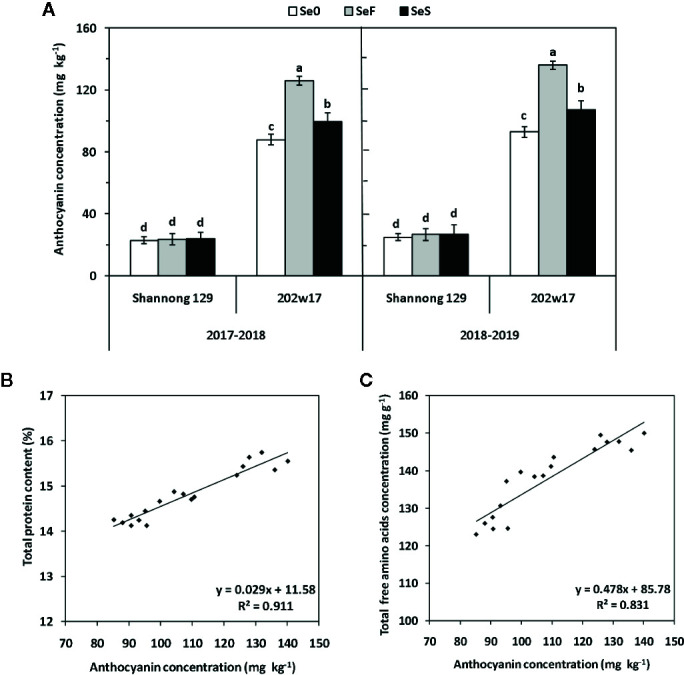
Effects of Se application on anthocyanin concentrations in the grain of wheat during two seasons. Anthocyanin concentrations **(A)**. The relationship between the SeMet concentration and total grain protein content **(B)** or the total concentration of free amino acids **(C)**. Se0, no Se control; SeF, foliar spray of Se; SeS, soil application of Se. Means and standard errors of three replicates are presented. The means are not significantly different within a given season when followed by the same lowercase letter using LSD at P < 0.05.

A low, basal concentration of anthocyanin (approximately 25 mg kg^−1^) was present in the grain of Shannong 129, and no significant difference was found among the three treatments of Se (**Figure 7A**). As expected, the concentration of anthocyanin was much higher in the grain of 202w17 than that of Shannong 129 in two seasons (**Figure 7A**). 202w17 with SeF had the highest anthocyanins (131 mg kg^−1^ on average in two seasons), which is 5.2-fold of that in Shannong 129 with SeF (**Figure 7A**). The anthocyanin concentration of 202w17 with SeF was 21.1% higher on average in two seasons than that of 202w17 with SeS, whereas the anthocyanin concentration of 202w17 with SeS was 12.6% higher on average in two seasons than that of 202w17 at Se0 (**Figure 7A**). The regression analysis of the grain samples of 202w17 showed that the anthocyanin concentration in grain is positively correlated with the total grain protein content (P < 0.01, **Figure 7B**) and the concentration of total free amino acids in grain (P < 0.01; **Figure 7C**), but not with grain weight (r = 0.1010, P > 0.05).

Two methods of Se application had different effects on the concentrations of mineral elements (**Tables 2** and **3**). The concentrations of Cr, Cd and Pd were significantly reduced in both methods of Se application (P < 0.05). For the concentrations of Mn and Cu, no significant difference was observed (P > 0.05). Regarding the Fe, Zn, Ca and Mg concentrations, significant increases was observed (P < 0.05). However, for these variables, SeF produced higher concentrations on average in two seasons than that SeS, phenomenon which was more obvious for 202w17.

**Table 2:** Effects of Se application on elements concentrations in the grain of wheat during two seasons.

Years	Cultivars		Heavy metal element (mg·kg^-1^)			Trace element (mg·kg^-1^)				Major element (mg·kg^-1^)	
			Cr	Cd	Pb	Fe	Mn	Cu	Zn	Ca	Mg
2017-2018	Shannong 129	Se0	0.48 b	0.04 a	0.06 c	10.49 c	34.03 a	7.58 a	24.95 d	79.99 c	1450.06 d
		SeF	0.47 c	0.03 c	0.04 d	27.68 ab	32.20 a	7.15 a	28.68 b	110.04 b	1823.34 b
		SeS	0.48 b	0.03 c	0.07 bc	29.94 a	33.18 a	7.18 a	27.13 c	90.66 c	1491.07 d
	202w17	Se0	0.49 a	0.05 a	0.11 a	28.32 ab	34.26 a	7.56 a	27.06 c	104.54 b	1512.02 d
		SeF	0.46 c	0.03 b	0.08 b	29.74 a	33.82 a	7.69 a	32.21 a	156.32 a	1899.42 a
		SeS	0.48 b	0.03 b	0.07 bc	24.74 b	32.38 a	7.78 a	28.18 b	110.34 b	1650.85 c
											
2018-2019	Shannong 129	Se0	0.48 a	0.05 a	0.06 cd	10.77 c	34.70 a	7.65 a	25.39 c	83.51 c	1456.02 d
		SeF	0.47 b	0.03 d	0.04 e	28.16 ab	33.20 a	7.21 a	28.86 b	111.37 b	1823.67 b
		SeS	0.48 a	0.03 d	0.06 d	29.69 a	32.18 a	7.28 a	27.47 b	91.31 c	1506.07 d
	202w17	Se0	0.49 a	0.05 b	0.11 a	28.37 ab	34.12 a	7.57 a	27.75 b	105.21 b	1515.35 d
		SeF	0.47 b	0.03 c	0.08 b	30.49 a	33.15 a	7.42 a	32.39 a	157.93 a	1897.55 a
		SeS	0.48 a	0.03 c	0.07 bc	26.07 b	31.71 a	7.26 a	28.54 b	111.34 b	1655.84 c

Se0, no Se control; SeF, foliar spray of Se; SeS, soil application of Se. The means are not significantly different within a given season when followed by the same lowercase letter using LSD at P < 0.05.

**Table 3:** F value and significance for protein, total free amino acids, anthocyanin, and elements.

Year	Source of variance	Protein	Total free amino acids	Anthocyanin	Toxic trace elements			Essential trace elements				Major element	
					Cr	Cd	Pb	Fe	Mn	Cu	Zn	Ca	Mg
2017-2018	Cultivar	78.58*	140.173**	53.25*	0.01	20.16*	4.86	0.52	0.24	3.56	9.63	13.61	10.57
	Fertilization method	91.48*	60.39*	1.05	18.02	37.45*	0.98	0.73	1.45	0.19	12.99	9.33	56.54*
	Cultivar×Fertilization method	1.40	0.95	564.25***	6.13*	0.76*	8.97**	113.51***	0.89	1.42	4.83*	26.34***	5.22*
													
2018-2019	Cultivar	80.34*	151.21**	49.39*	0.03	27.84*	12.44	0.74	0.78	0.16	10.67	11.77	11.32
	Fertilization method	106.62*	76.31*	1.14	20.72	57.47*	2.71	0.93	3.18	5.81	11.2	8.4	64.41*
	Cultivar×Fertilization method	1.26	0.25	716.15***	6.12*	1.78*	12.01**	179.50***	1.81	0.31	2.48*	33.87***	6.48*

*indicates a highly significant difference at P < 0.05; **indicates a highly significant difference at P < 0.01; ***indicates a highly significant difference at P < 0.001.

## Discussion

### Soil Application of Se Increases Plant Growth and Grain Yield of Wheat

Se has been considered as a non-essential for plants, but beneficial element under stress conditions (Jóźwiak and Politycka, 2019). Our results showed that soil application of Se could increase shoot dry weight and grain yield in both common and purple-grained wheat cultivars. Interestingly, such beneficial effect was not observed when foliar Se was applied at flowering (**Figure 1**). The difference between soil and foliar application of Se is the time of Se application and the plant part applied with Se. The soil application of Se was done before sowing, and the foliar spray of Se was at flowering. In the soil application, Se could be effective in the period from early growth of seedlings to plant maturity through Se uptake by roots. In contrast, the foliar application of Se could be effective only from flowering to plant maturity. The time difference suggests that the foliar application may have passed the critical stage of plant development at which a higher level of Se in plants could provide protection from the damage of oxidative stress (Lara et al., 2019). Notably, the increase in plant shoot dry weight and yield with Se application has also been reported in several other studies. Guerrero et al. (2014) showed that 10 µM selenate applied to wheat seedings increased plant biomass. Lara et al. (2019) reported that foliar spray application of 21 g Se ha^−1^ as selenate at grain filling significantly improved shoot biomass and grain yield in wheat. However, De Vita et al. (2017) found no effect of foliar application of selenate at the rates of up to 120 g Se ha^−1^ on wheat grain yield at the booting stage. There was no effect of soil application of up to 100 g Se ha^−1^ selenate at sowing on winter wheat yield in multiple sites, either (Broadley et al., 2010). The available data suggest that the beneficial effect of whether soil or foliar application of Se on plant growth and yield is dependent on timing of Se application and plant growth conditions. Therefore, to achieve both high yield and Se content in grain, foliar spray of Se may be required at the early growth stage before anthesis.

The increase observed in wheat shoot dry weight and grain yield could be due to the involvement of Se in the reduction of oxidative stress (Ahmad et al., 2016). Ascorbate peroxidase (ASP) and glutathione peroxidases (GSH-Px) detoxify hydrogen peroxide (H_2_O_2_), leading to the reduction inoxidative stress. The higher activity of ASP and constant levels of malonaldehyde and H_2_O_2_ in Se-treated wheat plants has shown to be favorable for photosynthesis under oxidative stress (Lara et al., 2019). In addition, Se could counteract oxidative stress by inhibiting lipid peroxidation, and increasing GSH-Px activity (Djanaguiraman et al., 2005). Therefore, the application of Se could reduce oxidative stress in wheat plants, leading to the increased biomass and yield. Clearly, further research is required to elucidate the underlying mechanisms.

### Higher Translocation of Se From Roots to Shoots and From Shoots to Grain in the Purple-Grained Wheat Cultivar Than in the Common Wheat Cultivar

When Se was applied either to soil or to leaves, the accumulation of Se was higher in the shoots and grain of 202w17 than that in Shannong 129 (**Figures 3A, B**), indicates that 202w17 can translocate more Se than Shannong 129 from roots to shoots *via* xylem and from shoots to grain *via* phloem. Foliar application of Se is transported from leaves to other parts of the plant through the phloem, whereas soil application of Se was transported from roots to shoots through the xylem. These results indicate that the effectiveness of foliar application of Se in wheat as a suitable strategy to increase Se concentrations in above-ground parts of the plant. The one possible reason was that xylem transport is more difficult than phloem transport (Zhu et al., 2009). The other possible reason was that the difference between soil and foliar application of Se is the time of Se application. A significant and positive correlation between grain Se concentration and the amount of precipitation during the growing season have been already indicated (Johnson, 1991). This result was in line with the general recommendation (application at GS-45 stage, at boots just swollen) given for humid regions (Rodrigo et al., 2014). Other authors have reported a better Se accumulation when Se was applied at flowering stage (Curtin et al., 2006) or even at grain filling (Chu et al., 2013) in humid regions or in irrigated crops. Hence, the foliar spray of Se was at flowering (May), which had a lot of precipitation may prompt differences in the uptake and accumulation of Se.

Genotypic variation in Se accumulation of grain has also been described in rice. Zhang et al. (2019) also found that a high-Se rice cultivar could transport more Se from roots to shoots. However, the underlying mechanism in the genotypic variation remains elusive. Phosphate transporters are involved in the uptake of selenite by wheat plants (Li et al., 2008). The over-expression of phosphate transporter OsPT2 significantly increases Se content in rice grain (Zhang et al., 2014), which indicates that phosphate transporters play a crucial role in Se retranslocation. Therefore, the higher expression of phosphate transporter genes in the purple-grained wheat, 202w17 may be responsible for the higher accumulation of Se in shoots and grain. Further experiments are needed to verify this hypothesis.

### Soil and Foliar Application of Se Increases the Levels of Organic Se, Protein, and Free Amino Acids in Both Common and Purple-Grained Wheat Cultivars

More than 90% of SeMet in crops can be absorbed by human body. Our results showed that the predominant form of organic Se in wheat grain was SeMet whether soil or foliar Se was applied, while SeCys2 was present as a minor form with less than 15.7% in total organic Se. Our finding agrees with the results of other studies on cereals such as wheat (Galinha et al., 2015). Our results also showed that the increase of SeMet by Se application was higher in the purple-grained wheat cultivar than the common wheat cultivar (**Figure 4**). Furthermore, the Se application had a positive effect on the content of total protein and total free amino acids in grain, in which the effects of foliar application of Se was significantly higher than those of soil application. This phenomenon was more obvious in purple-grained wheat cultivar (**Figure 5**). Interestingly, the SeMet concentration in grain is positively correlated with the levels of total protein and total free amino acids in grain (**Figure 5**). These results suggest that the levels of protein and free amino acids in grain contributing to the increased levels of organic Se in grain, whereas grain weight plays little role in the increased levels of organic Se in grain. This suggests that the levels of total protein and total free amino acids play a key in the accumulation of organic Se of grain. To further enhance the accumulation of organic Se in wheat grain, the increase in total protein and total free amino acids of grain is needed.

Nitrogen nutrients (protein and amino acids) are beneficial nutrients for human health, and also one of the important indicators for evaluating the nutritional quality of wheat. Nevertheless, it is also worth mentioning that Se is similar to sulfur (S) in chemical properties. Se can compete with S in the same metabolic pathway (Boldrin et al., 2016) and incorporated into amino acid in the form of SeMet. The concentration of Se in cells could affect the expression of key genes encoding S transporters, and the enzymes regulate S metabolism. This sometimes, but not always, results in increased concentrations of Cys and Met and S metabolites such as GSH and glucosinolates (Mostofa et al., 2017). Hence, the increased level of Se in plant tissues can affect N metabolism through its effects on S metabolism (Puccinelli et al., 2017). Therefore, the higher levels of total free amino acids and total proteins in grain resulted from the Se applications could be due to the impacts of Se on S metabolism.

### Soil and Foliar Application of Se Increases the Levels of Anthocyanin and Mineral Elements in Purple-Grained Wheat Cultivars

In the present study, we showed that the increased concentration of anthocyanin in the purple-grained wheat cultivar was more effective by foliar application of Se than by soil application of Se (**Figure 6A**). Similar results are obtained by Woch and Hawrylak-Nowak (2019) in alfalfa, radish, and white mustard sprouts applied with Se. In addition, we showed that the levels of total protein and total free amino acids were positively correlated with the anthocyanin concentrations in the grain of the purple-grained wheat cultivar (**Figures 6B, C**), but the dry weight of grain was not. The results suggest that the anthocyanin concentration in the purple-grained wheat, 202w17 is regulated by the metabolism of amino acid and protein, but not by carbon metabolism. This implies that the carbon skeleton is not a limiting factor in the biosynthesis of anthocyanins, but the altered N metabolism could have a link to the biosynthesis of anthocyanins. Many studies have shown that anthocyanin content of fruits and vegetables is closely related to the gene expression of the enzyme in anthocyanin biosynthesis pathways (Liu et al., 2013; Soubeyrand et al., 2014). Enzymes are functional proteins. An increase in the expression of the UFGT and F3H genes involved in the metabolism of anthocyanins, which results in an increase in the biosynthesis of these pigments, was shown in Se-treated lettuce plants (Liu et al., 2017). However, how Se applications enhance the biosynthesis of anthocyanins requires further investigation. As the color-grained wheat could provide a good dietary source of anthocyanin pigments (Abdel-Aal et al., 2016), and its production is sustainable. In this regard, the purple-grained wheat would be highly desirable for Se biofortification

Trace metal elements are widely presented in living tissue (Karavoltsos et al., 2020). Although some of them are also beneficial for human functional metabolism at low doses, excessive retention of any of toxic trace elements in the environment imposes threats to human health (Huang et al., 2008; Liang et al., 2020). The possible interactions and competition between Se and other major and trace elements are currently a key scientific issue (Feng et al., 2009). In the present study, we found that Se application reduced the concentrations of toxic trace elements (Cr, Cd and Pd) and increased essential trace elements (Fe and Zn) and major elements (Ca and Mg) in grains. However, foliar Se application provided higher Fe, Zn, Ca and Mg concentrations, and lower Cr, Cd and Pd concentrations in grains (**Table 2**). It was implied that the levels of Cr, Cd and Pd were possible antagonism correlated with the Se concentrations and the levels of Fe, Zn, Ca and Mg were possible antagonism correlated with the Se concentrations in the grain. Similar result are obtained by Liang et al. (2020), they found toxic trace elements (Cd, Pb) concentrations can be decreased by both methods (foliar or soil application). The reason may be that the combination of Se and Cr, Cd and Pd into insoluble complex, which promotes the precipitation and complexation of Se and restricts the bioavailability of Se. Ca and Mg presence is essential in the cell wall and, it is in charge of membrane integrity. Thus, the increase of Ca and Mg levels when Se application (Guerrero et al., 2014). Furthermore, Se may directly or indirectly influence the accumulation of micronutrients, such as Fe and Zn. Boldrin et al. (2013) also observed increase in Fe content with the foliar application of Se. Studies carried out by Zembala et al. (2010) with rape plant (*Brassica napus*) and wheat (*Triticum* spp.) shown that the application of Se increased Zn concentrations in the plant. In addition, the effect of Se on the absorption of trace elements in wheat depends not only on the method of Se application, but also on the cultivar of wheat. The two wheat varieties had significant differences in elements concentrations. Purple-grained wheat itself contains higher elements concentrations and the effect of Se application was more significant in purple-grained wheat. Therefore, based on our research data and previous results, inhibiting the heavy metal elements concentrations and increasing essential trace elements concentrations to reduce their harm to the human body or beneficial for human functional metabolism in agricultural crops. Se for fertilization programs is feasible.

## Conclusion

The soil application of Se could increase plant growth and grain yield in both common and purple-grained wheat cultivars, whereas the foliar application of Se was more effective in reducing Cr, Cd and Pd concentration and the increase of organic Se concentration, total protein content, total free amino acids and Fe, Zn, Ca and Mg in grain than the soil application. The purple-grained wheat 202w17 possessed not only a higher concentration of organic Se in grain, but also total protein, total free amino acids and Fe, Zn, Ca and Mg in grain than the common wheat Shannong 129 when Se was applied as foliar spray. In addition, the purple-grained wheat 202w17 had an enhanced level of anthocyanins with foliar spray of Se. Therefore, purple-grained wheat cultivars can be used as a good candidate for Se biofortification programs. Further studies are needed to examine more purple-grained wheat cultivars and timing of foliar spray for both high grain yield and organic Se content.

## Data Availability Statement

The original contributions presented in the study are included in the article/**Supplementary Material**; further inquiries can be directed to the corresponding author.

## Author Contributions

QX conceived and designed the experiments, performed the experiments, analyzed the data, prepared figures and/or tables, and approved the final draft. YS, XL, JC, and SK managed the field experiment. All authors discussed the results, and read and approved the final version of the manuscript. ZG, JW, and ZY conceived and designed the experiments, authored or reviewed drafts of the paper, and approved the final draft.

## Funding

This study was financially supported by the Key project of Shanxi Key R&D Program of China (201703D211001-02), Special Plan of Scientific Research for Shanxi Agriculture Valley of China (SXNGJSKYZX 201701), Shanxi Characteristic Wheat Industry Technology Innovative Strategic Alliance ([2020]17), Shanxi Scholarship Council of China from the Ministry of Finance, PR of China (2015-Key4), Shanxi Collaborative Innovation Centre with Featured Crops High-quality and Eﬃciency Production in Loess Plateau([2016]5), “1331 Project” Crop Ecology and Dry Cultivation Physiology Key Laboratory of Shanxi Province (201705D111007, [2017]14), “1331 Project” Organic Dry Farming and Cultivating Physiology Innovation Team Project of Shanxi Province ([2018]4), and Modern Agriculture Industry Technology System Construction (CARS-03-01-24).

## Conflict of Interest

The authors declare that the research was conducted in the absence of any commercial or financial relationships that could be construed as a potential conflict of interest.
