# Supplementary figures and images for: Methods of Selenium Application Differentially Modulate Plant Growth, Selenium Accumulation and Speciation, Protein, Anthocyanins and Concentrations of Mineral Elements in Purple-Grained Wheat
Source: Front Plant Sci. 2020 Jul 21;11:1114. doi: 10.3389/fpls.2020.01114 (PMC7396501; doi:10.3389/fpls.2020.01114)

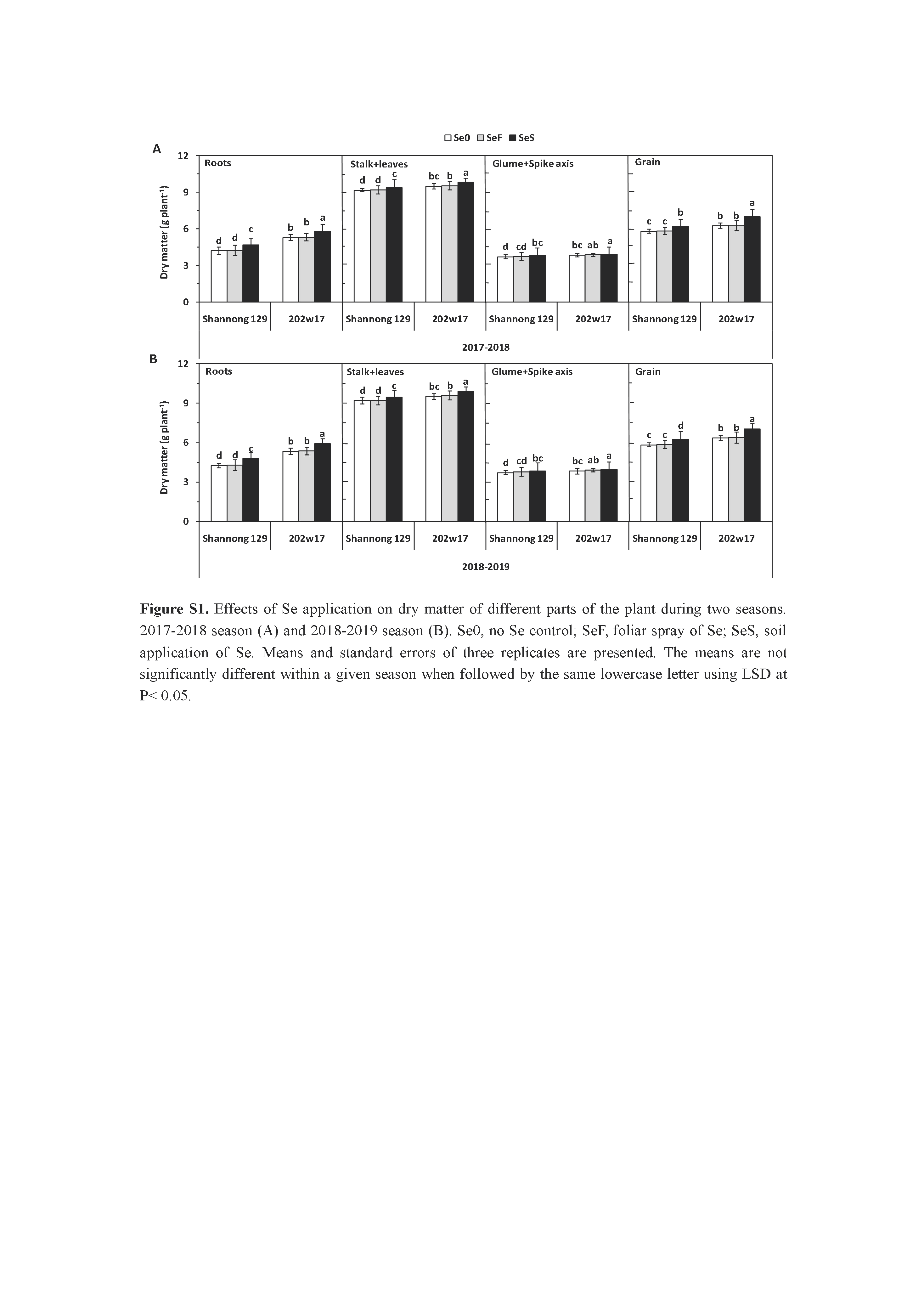

Supplement: Supplementary file 1 [file Image_1.tiff]

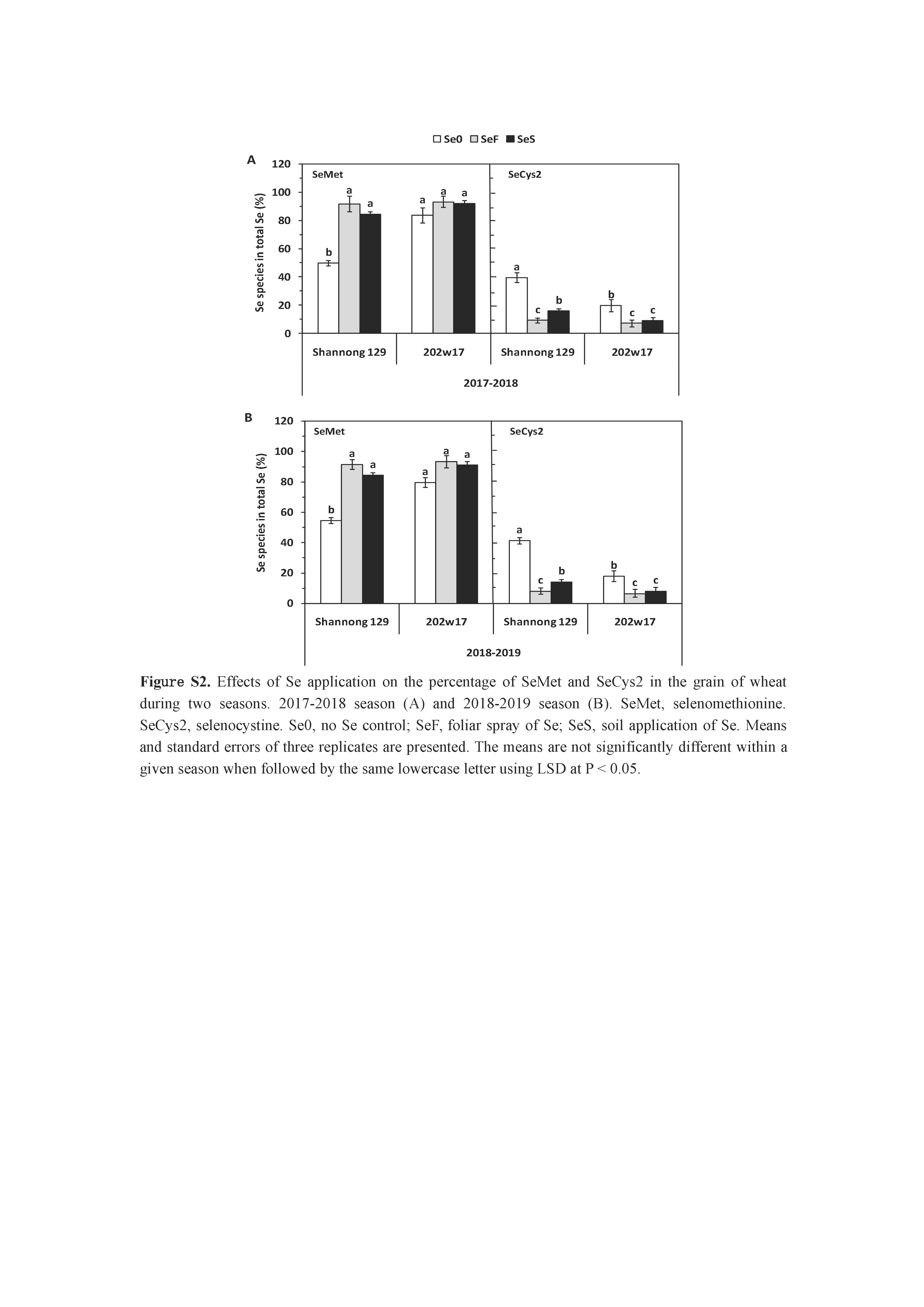

Supplement: Supplementary file 2 [file Image_2.tiff]
